# (−)-Loliolide Isolated from *Sargassum horneri* Protects against Fine Dust-Induced Oxidative Stress in Human Keratinocytes

**DOI:** 10.3390/antiox9060474

**Published:** 2020-06-02

**Authors:** Mawalle Kankanamge Hasitha Madhawa Dias, Dissanayaka Mudiyanselage Dinesh Madusanka, Eui Jeong Han, Min Ju Kim, You-Jin Jeon, Hyun-Soo Kim, Ilekuttige Priyan Shanura Fernando, Ginnae Ahn

**Affiliations:** 1 Department of Food Technology and Nutrition, Chonnam National University, Yeosu 59626, Korea; 198807@jnu.ac.kr (M.K.H.M.D.); 198793@jnu.ac.kr (D.M.D.M.); iosu5772@naver.com (E.J.H.); alswn1281@nate.com (M.J.K.); 2 Department of Marine Life Science, School of Marine Biomedical Sciences, Jeju National University, Jeju 63243, Korea; youjinj@jejunu.ac.kr (Y.-J.J.); gustn783@mabik.re.kr (H.-S.K.); 3 Marine Science Institute, Jeju National University, Jeju Self-Governing Province 63333, Korea; 4 National Marine Biodiversity Institute of Korea, 75, Jangsan-ro 101-gil, Janghang-eup, Seocheon 33662, Korea; 5 Department of Marine Bio-Food Sciences, Chonnam National University, Yeosu 59626, Korea

**Keywords:** *Sargassum horneri*, (–)-loliolide, fine dust, oxidative stress, HaCaT, apoptosis

## Abstract

The emergence of fine dust (FD) among air pollutants has taken a toll during the past few decades, and it has provided both controversy and a platform for open conversation amongst world powers for finding sustainable solutions and effective treatments for health issues. The present study emphasizes the protective effects of (–)-loliolide (HTT) isolated from *Sargassum horneri* against FD-induced oxidative stress in human HaCaT keratinocytes. The purification of (–)-loliolide was carried out by centrifugal partition chromatography. HTT did not show any cytotoxicity, and it further illustrated the potential to increase cell viability by reducing the reactive oxygen species (ROS) production in FD-stimulated keratinocytes. Furthermore, HTT suppressed FD-stimulated DNA damage and the formation of apoptotic bodies, and it reduced the population of cells in the sub-G_1_ apoptosis phase. FD-induced apoptosis was advancing through the mitochondria-mediated apoptosis pathway. The cytoprotective effects of the HTT against FD-stimulated oxidative damage is mediated through squaring the nuclear factor E2-related factor 2 (Nrf2)-mediated heme oxygenase-1 (HO-1) pathway, dose-dependently increasing HO-1 and NAD(P)H dehydrogenase (quinone) 1 (NQO1) levels in the cytosol while concomitantly improving the nuclear translocation of Nrf2. Future studies could implement the protective functionality of HTT in producing pharmaceuticals that utilize natural products and benefit the diseased.

## 1. Introduction

Recently, air pollution has become one of the most debated environmental issues throughout the globe. A real-time air quality index has been created to alert the public regarding levels of various air quality parameters, namely temperature, humidity, pressure, and pollutants such as nitric oxide (NO_2(g)_), sulfur dioxide (SO_2(g)_), and carbon monoxide (CO_(g)_) with immediate updates. This system is operational in many cities around the globe. Fine dust (FD) is one of the principal contributors to air pollution that is prominently observed in highly industrialized East Asian countries, including China, Japan, and Korea [1,2]. Recent studies have predicted increasing global warming in the upcoming decades, fueled with various deleterious anthropogenic activities that can drastically catalyze the desertification process, which escalates the overall dust emissions from the desert areas and consequently affects environmental dynamics and ecosystems [3]. Strong winds from the northern and northwestern parts of the arid and semi-arid regions of Mongolia and China carry particulate matter as far as the North American continent that can be identified from the mineral signature of the aerosol composition [3,4]. The overexploited use of coal and petroleum for energy has become a major contributor to the emerging FD pollution with unburnt particles constantly being released into the atmosphere [2]. According to Raloff (2001), FD particles originating from industries may contain unburnt hydrocarbons, soot, CO_(g)_, carcinogens (asbestos, pesticides, and silica), and heavy metals such as cadmium, mercury, led, chromium, arsenic, and copper [5]. The above environmental hazards can cause various detrimental effects on human health, triggering allergic reactions and oxidative stress. Chronic exposure would cause the development of conjunctivitis, asthma, rhinitis, and dermatitis [6,7]. In some instances, intercontinental FD clouds can also harbor pathogenic bacteria and viruses [5]. Though numerous studies have been carried out to understand the health impacts of FD, the available literature regarding the effects of FD-induced oxidative stress in human keratinocytes is insufficient.

The consumption of marine algae in East Asian countries has increased over the last few decades with the identification of a large number of bioactive natural products that benefit human health [8,9]. *Sargassum horneri* is an edible brown alga that is abundant along the coasts of China, South Korea, Japan, and the North American continent [10,11]. Due to its high biomass and nutritional value (as it is packed with vitamins, polysaccharides, amino acids, and dietary fibers), it is regarded as one of the delicacy dishes in Korea. Moreover, for centuries, *S. horneri* has been used as an ingredient in indigenous medicine [10]. Various studies have addressed the beneficial effects of bioactive compounds such as sargachromenol, phenolics, fucoxanthin, phlorotannins, proteoglycan, and sulfated polysaccharides isolated from *S. horneri* [7,8,9,10,12].

(–)-Loliolide ((6S,7aR)-6-hydroxy-4,4,7a-trimethyl-5,6,7,7a-tetrahydro-1-benzofuran-2(4H)-one) is a frequently available monoterpenoid lactone. First discovered in English Ryegrass (*Lolium perenne*) in 1964, (–)-loliolide (HTT) has since been found in many plants and animals in both terrestrial and marine ecosystems [13]. Various biological functions of HTT have been reported, including antioxidant, anti-fungal, antibacterial, and anti-cancer activities; in some instances, it has been used as an alternative medicine for depression and diabetes [13,14]. The major emphasis of the current study was to demonstrate that FD has the ability to induce keratinocytes to produce reactive oxygen species (ROS), inevitably resulting in oxidative stress that causes cell damage and apoptosis; another emphasis was the potential protective effects of HTT in developing future pharmaceuticals and cosmeceuticals.

## 2. Materials and Methods

### 2.1. Raw Materials, Chemicals, and Reagents

Urban aerosols (NIES CRM No. 28) were acquired from the National Institute for Environmental Studies (Tsukuba, Ibaraki, Japan). TrytonX-100, 3-(4-50dimethyl-2yl)-2-5-diphynyltetrasolium bromide (MTT), 2′,7′-dichlorofluorescin diacetate (DCFH-DA), dimethyl sulfoxide (DMSO) ethidium bromide, and low melting agarose were purchased from Sigma-Aldrich (ST. Louis, MO, USA). Human HaCaT keratinocytes were donated by the American Type Culture Collection (Manassas, VA, USA). Dulbecco’s Modified Eagle Medium (DMEM) and antibiotics (streptomycin and penicillin) were purchased from GibcoBRL (Grand Island, NY, USA), and the fetal bovine serum (FBS) was obtained from Welgene (Gyeongsangbuk-do, South Korea). Relevant antibodies were purchased from Cell Signaling Technology Inc. (Beverly, MA, USA) and Santa Cruz Biotechnology Inc. (Dallas, TX, USA). All additional chemicals and reagents used were purchased from commercial sources with the highest quality.

### 2.2. Sample Preparation and Isolation of HTT

The collection of *S. horneri* samples was done during the spring season in 2015 along Jeju Island coasts in South Korea. Samples were washed with tap water to remove excess salts and other contaminants, and this process was followed by storage under refrigerated conditions. A detailed extraction and purification method of HTT was described in our previous publication [15]. Briefly, *S. horneri* dry powder was extracted with 80% methanol at 37 °C and concentrated using a rotary evaporator. The crude extract was sequentially fractioned into n-hexane, chloroform (CMSH), and ethyl acetate. CMSH was further purified by a high-performance centrifugal partition chromatography system (Sanki Engineering, Kyoto, Japan) using upper and lower phases of an equilibrated solvent system composed of n-hexane/ethyl acetate/methanol/water (5:5:5:5, *v*/*v*). The separation was monitored at 240 nm by an L-4000 UV detector (Hitachi, Japan). The eluant was collected into test tubes using a fraction collector (FC 203B, Gilson, South Korea). The further purification of the active fraction was carried out by a Prep HPLC system (Waters, Milford, MA, USA) using a semi-preparative C18 (YMC-Pack ODS-A, 5 μm, 10 × 250 mm) column. The gradient elution was carried out using acetonitrile: distilled water as; 0–60 min 5:95–100: 0 *v*/*v*; 60–70 min 100:0–100: 0 *v*/*v* at a flow rate of 3 mL min^−1^, while the UV absorbance was observed at 230 and 254 nm by a Waters 2998 photodiode array detector (Waters, Milford, MA, USA).

### 2.3. In Vitro Analysis

#### 2.3.1. Cell Culture and Maintenance

HaCaT (human keratinocyte) cells were cultured using DMEM media containing 10% (*v*/*v*) heat-inactivated FBS and 1% (*v*/*v*) streptomycin and penicillin (100 μg mL^−1^). Cells were maintained inside a controlled environment with 5% CO_2_ at 37 °C. Periodic subculturing was practiced, and cells with exponential growth were used for experiments that achieved a 95% confluency. HTT was initially dissolved in DMSO and diluted using DMEM for cell culture experiments. The final concentration of DMSO in treated samples was kept below 0.1%. DMEM was added to control cell groups to maintain a constant volume.

#### 2.3.2. Cell Viability and ROS Production

The concentration of FD used, exposure time, and the method were based on previous observations and preliminary experiments [7]. Initially, FD was suspended in DMEM, followed by sonication for one minute to separate any clumps. FD suspended solvent was vortexed prior to each treatment. HaCaT cells were seeded at a concentration of 1 × 10^5^ cells mL^−1^ in 96 well plates, with 2 × 10^4^ cells in each well, which were incubated for 24 h. Different concentrations of HTT were used to treat the cells, and after one hour, cells were stimulated with FD (150 μg mL^−1^) for one-hour. The FD-containing media were carefully removed and washed three times, followed by adding new media. MTT and DCFH-DA assays were conducted to measure the cell viability and intracellular ROS generation, respectively [16].

Simultaneously, the same concentration of cells was seeded in a 24 well plate and 6 cm culture dishes with 5 × 10^4^ cells per well and 3 × 10^5^ cells per dish for the respective detection of ROS levels by using fluorescence microscopy and flow cytometry under DCFH-DA staining. The side scatter vs. forward scatter (SSC vs. FSC) gating strategy was used in the flow cytometry to eliminate cell debris. Images of the fluorescence microscopy were obtained by using an Invitrogen™ EVOS™ Auto 2 fluorescence microscope (Thermo Fisher Scientific, Bothell, WA, USA), and flow cytometry analysis was conducted using a Beckman Coulter CytoFLEX system (Beckman Coulter, Brea, CA, USA).

#### 2.3.3. Nuclear Morphological Analysis

Nuclear staining was carried out by using Hochest 33342 following the method described by Yang et al. (2011) with slight modifications [14]. HTT treatment and FD stimulation followed the same method mentioned in Section 2.3.2. After washing with phosphate-buffered saline (PBS), the Hoechst 33342 reagent was added, achieving a final concentration of 2 μg mL^−1^ per well, each of which were incubated for 20 min at 37 °C in the dark. The visualization of nuclear morphology was carried out using a fluorescence microscope.

#### 2.3.4. Annexin V Assay

Early apoptosis detection was carried out by flow cytometry using an eBioscience ™ Annexin V Apoptosis Detection Kit (Thermo Fisher Scientific, Carlsbad, CA, USA) according to the manufacture’s guidelines. HTT-treated, FD-induced cells were incubated for six hours prior to harvesting for the assay.

#### 2.3.5. Cell Cycle Analysis

Cell cycle analysis was conducted according to a pre-described method by Fernando et al. (2017) with slight alterations [17]. Briefly, HTT-treated and FD-induced cells, as described in Section 2.3.2, were used. Cell harvesting was carried out 24 h after the incubation, which was followed by fixing in 70% ethanol at 4 °C. Cells were washed using PBS, treated with 2 mM of ethylenediaminetetraacetic acid (EDTA), and resuspended in 300 µL of PBS–EDTA-containing RNase (0.2 μg mL^−1^) and propidium iodide (50 μg mL^−1^) for 30 min. The analysis was conducted using a flow cytometer.

#### 2.3.6. Alkaline Comet Assay

The alkaline comet assay was carried out following the procedure by Fernando et al. (2017) with minor modifications [17]. The sample treated cells were incubated for 24 h and dispersed in 1% low melting agarose at 40 °C. The cell-agarose mixture was pipetted onto agarose pre-coated microscopic slides and rested for solidification. Then, the slides were lysed overnight by submerging in a 1% TrytonX-100-containing lysis buffer at 4 °C. Electrophoresis was conducted for 30 min at 30 V/300 mA, while the temperature was maintained at 4 °C. A neutralizing buffer was used to wash the slides three times, prior to staining with ethidium bromide (2 μg mL^−1^) for five minutes. Pre-chilled water was used to wash away any excess dye, and the visualization was conducted using a fluorescence microscope.

#### 2.3.7. Western Blot Analysis

As elucidated in Section 2.3.2, HTT-treated FD-induced cells were harvested and lysed using a NE-PER^®^ nuclear and cytoplasmic extraction kit and radioimmunoprecipitation assay (RIPA) buffer (Thermo Scientific, Rockford, IL, USA). Isolated cytoplasmic and nuclear protein levels were determined by using Bio-Rad Protein Assay Dye (Bio-Rad Laboratories, Inc., Hercules, CA, USA), and a concentration gradient of bovine serum albumin was used as the reference standard. A normalized amount of protein was loaded into 10% polyacrylamide gels and electrophoresed. After transferring the protein bands to nitrocellulose membranes, blocking was carried out with 5% skim milk in tris buffered saline (TBS) containing Tween-20 for two hours. Primary antibodies were added to the membranes and incubated overnight at 4 °C with continuous agitation. Then, the membranes were incubated with their respective enzyme-linked secondary antibodies for two hours at room temperature and visualized using SuperSignal™ West Femto Maximum Sensitivity Substrate (Thermo Scientific Inc., Rockford, IL, USA) on a Core Bio DavinchChemi imager (Seoul, Korea).

### 2.4. Statistical Analysis

PASW Statistics 18 was used to conduct the statistical analysis, and data are expressed as mean ± standard error of the mean (SEM). The significant differences amongst data sets were determined by using the ANOVA with the Duncan’s multiple range test. The significance of data was determined at *p* < 0.05.

## 3. Results

### 3.1. Effect of HTT on Cytotoxicity, Cell Viability and Intracellular ROS Production of FD-Induced HaCaT Cells

**Figure 1:**
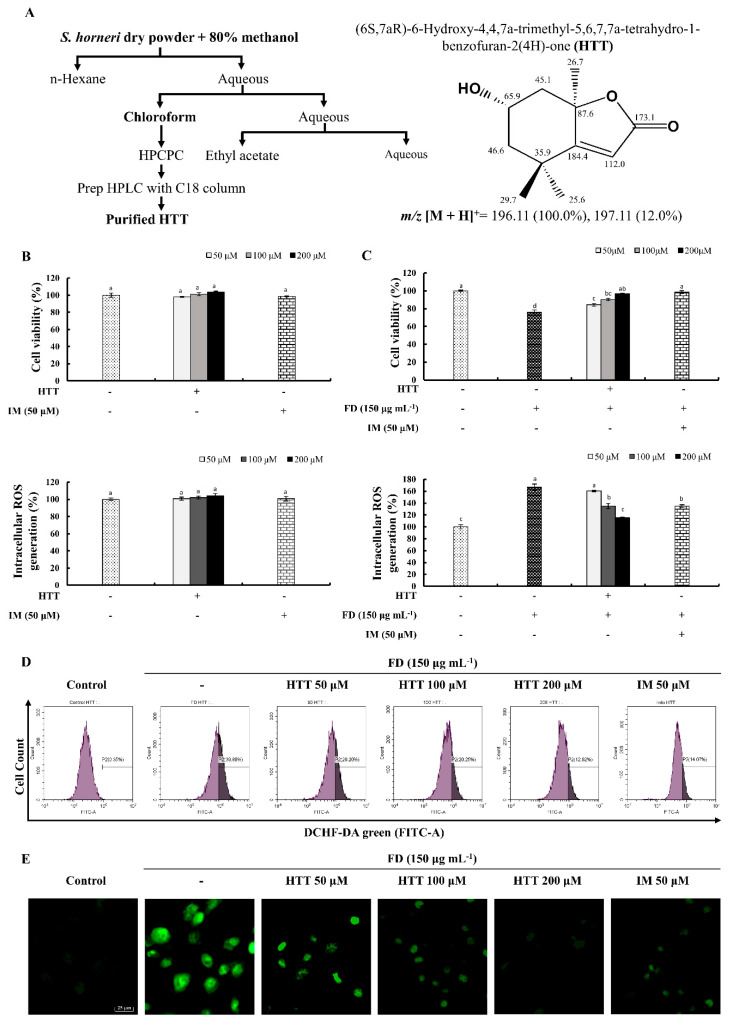
(**A**) Purification process and the chemical structure of (–)-loliolide (HTT) with ^1^H NMR chemical shifts. The effects of HTT on cell viability and intracellular reactive oxygen species (ROS) production (**B**) in the absence of fine dust (FD) and (**C**) with FD-stimulated HaCaT cells. Analysis of ROS levels in 2′,7′-dichlorofluorescin diacetate (DCFH-DA)-stained HaCaT cells by (**D**) flow cytometry and (**E**) fluorescence microscopy. Seeded cells were pre-treated with different concentrations of HTT (50–200 μM) at one hour prior to stimulation with 150 μg mL^−1^ of FD. Indomethacin (IM) was used as the positive control. The experiments were conducted in three independent determinations (*n* = 3), and the values are given as means ± SEM. Error bars with different letters are significantly different (*p* < 0.05).

The extraction of *S. horneri* and further purification that led to the isolation of HTT is depicted in Figure 1A. Details of this isolation procedure were described in our previous publication [15]. According to Figure 1B, a non-significant increment of cell viability and ROS was observed, suggesting that the used concentrations of HTT had no cytotoxicity towards HaCaT cells. FD had induced the production of intracellular ROS compared to the control cell group while simultaneously reducing cell viability. However, treating cells with different concentrations of HTT significantly increased the cell viability compared to the cells that were exposed only to FD, concomitantly minimizing the production of intracellular ROS in a dose-dependent manner (Figure 1C). The highest concentration of HTT (200 μM) showed a similar cell viability activity to those of the positive control, indomethacin (IM). The protective effects of HTT against FD-induced ROS generation were confirmed by flow cytometry and fluorescence microscopic analysis (Figure 1D,E). FD-stimulated cells indicated a rightward shift of the cell population with a higher intensity of fluorescence compared to the control cells. The green fluorescence in Figure 1E was at its peak for the FD-stimulated cell group compared to the control. In both instances, treating with HTT dose-dependently reduced the fluorescence of DCFH-DA, confirming the protective effects of HTT against FD-induced oxidative stress.

### 3.2. HTT Inhibited Early Apoptosis and Apoptotic Body Formation in FD-Induced HaCaT Cells

**Figure 2:**
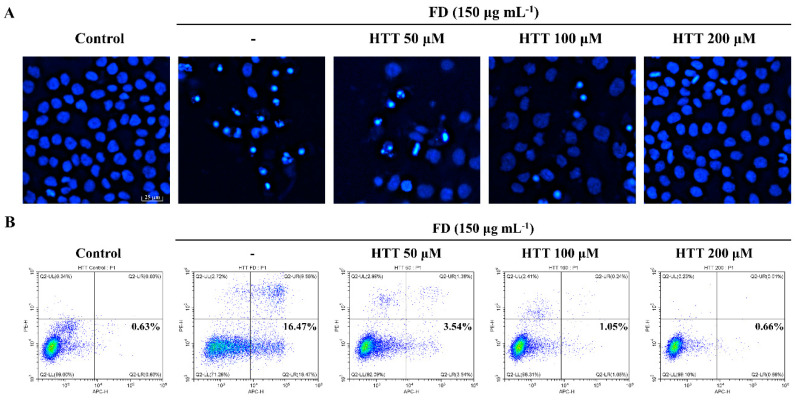
The effect of (–)-loliolide (HTT) on apoptotic body formation and early apoptosis in fine dust (FD)-induced HaCaT cells. Fluorescence microscopy of nuclear morphological analysis using (**A**) Hoechst 33342 dye and early apoptosis detection by (**B**) annexin V with flow cytometry. HTT pre-treated (50–200 μM) exposed to FD for one hour before replenishing with new media. The experiments were conducted in triplicates with three independent trials to confirm repeatability.

Phosphatidylserine (PT) is an inner plasma membrane phospholipid that becomes translocated to the outer plasma membrane during the early stages of apoptosis. Annexin V is a phospholipid-binding protein that has a high affinity towards PT, and that can be utilized to detect early apoptotic cells [18]. The Hoechst 33342 nuclear staining is a unique fluorescent staining technique used to visualize apoptotic bodies and thereby determine the level of nuclear condensation and DNA damage [17]. According to the results of Figure 2A, HaCaT cells stimulated by FD indicated an apparent reduction of total cell density, with a notable increment of apoptotic bodies compared to the control; this indicated nuclear condensation. Cells that were pre-treated with HTT (200 μM) had the least amount of apoptotic bodies and the highest cell population compared to the rest of the treatment groups. Compared to the control, FD-induction increased the cell population in early apoptosis from 0.63% to 16.47%, as depicted in the lower right quadrants of the histograms in Figure 2B. The dose-dependent reduction of early apoptotic cell population further confirmed the protective effects of HTT against FD-induced apoptosis.

### 3.3. HTT Attenuates DNA Damage and Apoptotic Cells in the Sub-G_1_ Phase

**Figure 3:**
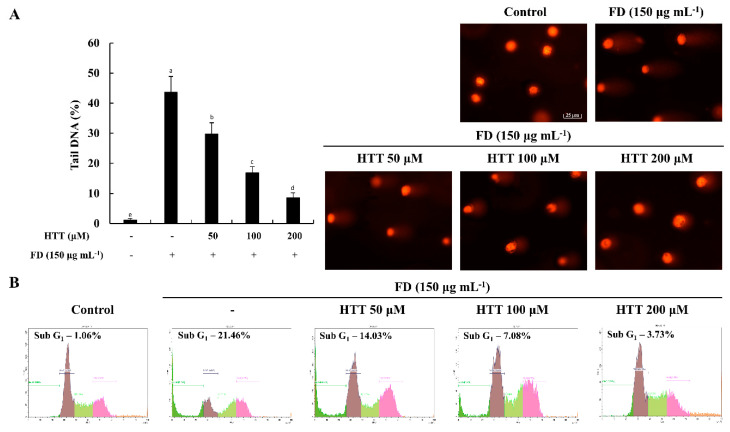
(–)-loliolide (HTT) inhibited DNA breakage and apoptosis in fine dust (FD)-stimulated HaCaT cells. (**A**) Comet tails and (**B**) sub-G_1_ cell populations of FD-stimulated HaCaT cells treated with HTT. Cells were treated with different concentrations of HTT (50–200 μM) prior to stimulation with FD. Comet tail DNA percentages were analyzed using the OpenComet plugin of the ImageJ software. Results were analyzed as three independent determinations (*n* = 3) to confirm repeatability. Values are given as means ± SEM, and error bars representing different letters are significantly different (*p* < 0.05).

The alkaline comet assay—also known as single-cell gel electrophoresis—is a widely used method to detect DNA damage in cells. The comet tail length and tail DNA content are considered to be proportional to DNA damage [17]. As shown in Figure 3A, the tail DNA content drastically increased in the FD-treated cells compared to the control cell group, while the HTT pre-treatment reduced the tail DNA content in a dose-dependent manner suggesting the potential ability of HTT to reduce DNA damage induced by FD. Furthermore, as illustrated in Figure 3B, a significant proportion of FD-induced untreated cells were in the sub-G_1_ phase (21.46%) compared to the control cell group (1.06%). However, the sub-G_1_ cell population was dose-dependently reduced with HTT treatment. These results coincided with the results obtained for the Hoechst 33342 nuclear staining, further establishing the protective effect of HTT against FD-induced DNA damage in HaCaT cells.

### 3.4. HTT Attenuates Apoptosis via the Mitochondrial Pathway

**Figure 4:**
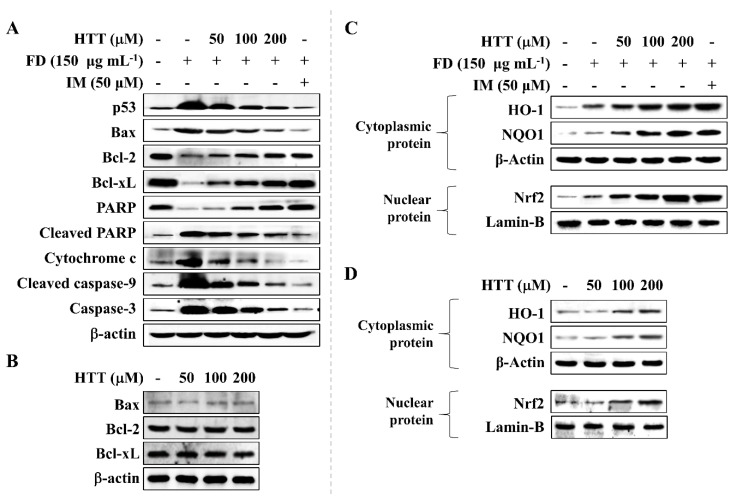
Effect of (–)-loliolide (HTT) on the levels of key apoptosis and antioxidant molecular mediators. Effect of HTT on key apoptotic mediators in (**A**) fine dust (FD)-induced and (**B**) non-induced cells. Effect of HTT on nuclear factor E2-related factor 2 (Nrf2)/heme oxygenase-1 (HO-1) signaling molecules in (**C**) FD-induced and (**D**) non-induced HaCaT cells. Seeded cells were treated with different concentrations of HTT one hour before stimulation. Indomethacin (IM) was used as the positive control. The experiment was done in three independent trials to confirm repeatability.

The effects of HTT on apoptosis-related proteins were analyzed using Western blotting. According to the results shown in Figure 4A, stimulating cells with FD increased the levels of pro-apoptotic proteins p53, B-cell lymphoma 2-associated X protein (Bax), cleaved caspase-9, cytochrome c, cleaved poly (ADP-ribose) polymerase (PARP), and caspase-3 while reducing the anti-apoptotic proteins B-cell lymphoma 2 (Bcl-2), PARP, and B-cell lymphoma extra-large (Bcl-xL) compared to the control cell group. Nonetheless, the HTT pre-treated cells showed increased levels of Bcl-2, Bcl-xL, and PARP while simultaneously suppressing the levels of Bax, cleaved PARP, cleaved caspase-9, caspase-3, cytochrome c, and p53 in a concentration-dependent manner that suggested the protective effect of HTT against FD-induced apoptosis in HaCaT cells. Furthermore, according to Figure 4B, treating cells with HTT (without FD stimulation) had no effect on apoptotic related protein levels (Bax, Bcl-2, and Bcl-xL) in HaCaT cells.

### 3.5. Effect of HTT on Upregulation of Nrf2/HO-1 Pathway Proteins

Figure 4C illustrates nuclear factor E2-related factor 2 (Nrf2) pathway-related protein level expressions. FD slightly increased the nuclear translocation of Nrf2. This was consistent with the increment of NAD(P)H dehydrogenase (quinone) 1 (NQO1) and heme oxygenase 1 (HO-1) protein levels in the cytosol compared to the control cells. However, the HTT pre-treated cells had gradually increased levels of NQO1, HO-1, and Nrf2 dose-dependently. Moreover, the levels of NQO1, HO-1, and Nrf2 had a slight dose-dependent increment without FD-stimulation, thus suggesting the potential of HTT to activate the Nrf2/HO-1 antioxidative pathway (Figure 4D). Indomethacin was used as a positive control in the experiment for the comparison of its effects with HTT.

## 4. Discussion

“Oxidative stress” and “ROS” are two frequently used terms that complement each other and play major roles in human health [19,20]. Weakened antioxidant defense mechanisms, coupled with excess production of ROS such as hydrogen peroxide (H_2_O_2_), superoxide anion radicals (O_2_^∙−^), hydroxyl radicals (^∙^OH), hypochlorite (ClO^−^), and nitric oxide radicals (NO_x_^∙^), is considered as primary causes of oxidative stress that damages major macromolecules of cells such as carbohydrates, proteins, lipids, and DNA, ultimately leading to cellular death. Moreover, the above incidents may trigger diseases such as diabetes, atherosclerosis, various neurodegenerative and cardiovascular disorders, and even cancer [19,21].

FD is one of the highly discussed issues in the modern world, as it affects countries all around the globe. Prolonged exposure to FD-polluted air containing various toxic substances, namely heavy metals and organic pollutants such as polycyclic aromatic hydrocarbons (PAHs) can synthesize ROS (e.g., the generation of HO^•^ via Fenton’s reaction) inside the human body, subsequently leading to pulmonary and systemic oxidative stress [22,23,24]. According to Mori et al. (2008), the FD used in the current study mainly consisted of Si (14.9%), C (12%), Ca (6.69%), Al (5.04%), S (3.91%), Fe (2.92%), Mg (1.40%), K (1.37%), Cl (0.807%), Na (0.796%), N (0.79%), Ti (0.292%), P (0.145%), and Zn (0.114%), as well as minor amounts of other elements and substances including PAHs such as fluoranthene, benzo (b) fluoranthene, pyrene, indeno (1,3,3,-cd) pyrene, benzo (ghi) perylene, benz (a) anthracene, benzo (k) fluoranthene, and benzo (a) pyrene—all of which have the potential to induce oxidative stress in human keratinocytes [25]. Furthermore, FD reported from other heavily industrialized areas has shown similar general constituents (elements and PAHs) [26,27].

HTT is a crucial bio-active compound available in *S. horneri* that possesses potent medicinal properties and has been used as folk medicine in countries such as Japan, Mexico, Egypt, and the Philippines [13]. Therefore, the potential therapeutic use of HTT in attenuating FD-induced oxidative stress was investigated in the current study.

The HaCaT cell model is commonly used in dermatological studies to represent typical human keratinocytes. Located on the epithelium of the outer skin, these cells are prime candidates that have direct contact with FD and the potential to induce oxidative damage. This was further confirmed from the results shown in Figure 1C–E, where the concentration of the used FD increased the generation of ROS in HaCaT cells, thus increasing oxidative damage and inevitably reducing cell viability. However, the used concentrations of HTT increased the cell viability while minimizing the overall ROS production without any cytotoxicity, thus further illustrating the potential of HTT in attenuating the oxidative stress generated from exposure to FD. Further evidence was acquired from flow cytometry and fluorescence microscopy using DCFH-DA staining, which indicated the reduction of ROS levels upon HTT treatment. The flowcytometric analysis allows to omit the florescence incidents from dead cells and cell debris. The above results, together with fluorescence microscopy, confirmed the ability of HTT to attenuate oxidative stress caused by FD.

Apoptosis is considered to be the major outcome in cells and tissues that are regularly exposed to high levels of oxidative damage [19,21]. DNA fragmentation and nuclear condensation, along with the formation of apoptotic bodies, is a crucial identification criterion of controlled cell death [28]. Hoechst 33342 staining is a widely used staining technique that is implemented to visualize the formation of apoptotic bodies. The annexin V assay has the capability to identify different stages of apoptosis in particular cell lines and to provide the foundation to detect and differentiate between viable, early, and late apoptotic cells [18]. Based on the obtained results, HTT minimized the apoptotic body formation, an effect that was further confirmed by the results of the annexin V and alkaline comet assays. The cell cycle analysis histograms gave clear evidence of the ability of HTT on attenuating apoptosis from the reduction of sub-G_1_ hypodiploid subpopulations in the cell cycle [29].

The mitochondrial-mediated apoptosis pathway is a highly complex cascade of reactions that regulates cell death. Bcl-2 family proteins, including pro-apoptotic proteins such as BH3 interacting-domain death agonist (Bid), Bcl-2-associated death promoter (Bad), Bcl-2 related ovarian killer (Bok), Bcl-2 homologous antagonist killer (Bak), Bcl-2-interacting killer (Bik), Bcl-2-modifying factor (Bmf), Bim, Nova, p53 upregulated modulator of apoptosis (Puma) and Bax; anti-apoptotic proteins (Bcl-xL, Bcl-2, and Mcl-1) coupled with caspases regulate the complex procedure [17,21]. The activation of the mitochondrial-mediated apoptosis pathway takes place with the activation of p53 and pro-apoptotic Bcl-2 family proteins in the cytosol, which leads to the inhibition of anti-apoptotic Bcl-2 family proteins localized on the mitochondria’s outer membrane. This phenomenon increases the mitochondrial outer membrane permeabilization, which leads to the release of apoptosis-promoting proteins cytochrome c, endonuclease G, and the apoptosis-inducing factor to the cytosol. The combination of procaspase-9, apoptosis activating factor-1, and cytochrome c are known as the apoptosome. This complex is the prime candidate for activating caspase-9, which enables a cascade of reactions to take place activating caspases-3, -6, and -7 that further continue the apoptosis process [21]. Moreover, initiator caspases, namely caspase-2, -8, -9, and -10 are responsible for the apoptosis initiation and execution, while the effector caspases such as caspase-3, -6, and -7 are key intermediates that connect the cascade. This leads to the rise of morphological and biochemical modifications in key regulatory molecules such as PARP, which is a protein responsible for the repairing ability, transcription, and stability of DNA [17]. Results observed in the present study showed that HaCaT cells exposed to FD demonstrated apoptosis via the mitochondrial-mediated apoptosis pathway, and HTT dose-dependently minimized the pro-apoptotic molecule levels in cells. Furthermore, an in-depth analysis of mRNA expressions of key mediators related to the mitochondrial-mediated apoptosis pathway could be implemented in future studies and could be utilized to provide a thorough insight into the mechanism.

The activation of the Nrf2 pathway is considered to be one of the key regulatory processes that enable cells to exhibit resistance against redox imbalances and oxidative stress [30]. Inactive Nrf2 is localized in the cytosol bound with Kelch-like ECH-associated protein 1 (Keap1) that inhibits the activation of Nrf2 [31]. With the ongoing oxidative stress conditions in the cell, the Keap1 protein is inactivated by an upstream molecule known as p62 and promotes the nuclear translocation of activated Nrf2. This phenomenon is the key inducer of activating NQO1—a drug-metabolizing enzyme—and HO-1, which triggers protective effects by regulating redox imbalance. Nevertheless, the oxidative stress caused by the stimulant overwhelms the protective effects of antioxidative gene HO-1, limiting its potential ability to maintain redox imbalance [31]. However, based on the results, HTT potentially increased the Nrf2 levels in the nucleus, simultaneously increasing the HO-1 and NQO1 proteins in a concentration-dependent manner in the cytosol, suggesting that the cytoprotective effects of HTT against FD-induced oxidative damage was carried via the Nrf2/HO-1 signaling pathway.

## 5. Conclusions

Considering the results of the current study, HTT holds potent protective competence in attenuating FD-induced oxidative stress in human keratinocytes. Furthermore, new techniques and methods could be materialized to identify and increase the efficiency of extracting HTT like bio-active compounds from *S. horneri*. Future studies could be implemented on applications of HTT-based pharmaceutical and cosmetic products as a means of maintaining healthy skin.
